# Clinicians’ perspectives on and interest in participating in a clinical data research network across the Southeastern United States

**DOI:** 10.1186/s12913-018-3399-9

**Published:** 2018-07-20

**Authors:** Kim M. Unertl, Alecia M. Fair, Jacquelyn S. Favours, Rowena J. Dolor, Duane Smoot, Consuelo H. Wilkins

**Affiliations:** 1 Department of Biomedical Informatics, Vanderbilt University School of Medicine, Nashville, TN 37203 USA; 2 Vanderbilt School of Medicine and the Meharry-Vanderbilt Alliance, Nashville, TN 37208 USA; 3 Division of General Internal Medicine, Department of Medicine, Duke University School of Medicine, Durham, NC USA; 4 Department of Internal Medicine, Meharry Medical College, Nashville, TN 37208 USA; 5 Vanderbilt University Medical Center, Nashville, TN 37208 USA

**Keywords:** Qualitative research, Clinicians, Clinical data research network

## Abstract

**Background:**

Partnerships between clinicians and researchers could increase the generalizability of research findings and increase uptake of research results across populations. Yet engaging clinicians in research is challenging. Clinical Data Research Networks (CDRNs) provide access to a broad array of clinical data, patients, clinicians and health systems by building on existing health records (EHRs) to facilitate multi-site community engaged research (CEnR).

**Methods:**

A mixed-methods sequential explanatory design was employed. Sixty semi-structured interviews with clinicians from various disciplines and healthcare settings were conducted using five open-ended questions. Inductive content analysis was used to identify emerging themes in the data.

**Results:**

We identified the following emerging themes: 1) Research with relevance and benefits to clinics and provider’s patient population; 2) Difficulties of engaging in research with existing patient care demands; 3) Clear and continuous two-way communication about research, coordinated with provider and clinic needs; 4) Tailored compensation approaches meet provider preferences; 5) Increasing clinician awareness about Clinical Data Research Networks (CDRNs).

**Conclusion:**

Our interview study provides insight into community clinician perspectives on Clinical Data Research Networks, indicating motivations and challenges to research involvement including consequences of time spent on research participation, barriers to expanding research and meaningful involvement in research governance. Findings can be used to guide the development of strategies to better engage providers in research in clinical settings, which could ultimately improve patient outcomes.

**Electronic supplementary material:**

The online version of this article (10.1186/s12913-018-3399-9) contains supplementary material, which is available to authorized users.

## Background

Authentic stakeholder involvement is critical to informed health care decisions and implementing patient-centered outcomes research, Significant stakeholder engagement begins before the initial study design, leveraging stakeholders’ interests continuously throughout the life of the research [1]. Approaches to increase involvement of patients in clinical research, such as Practice-Based Research Networks (PBRNs), have a lengthy history in the United States [2].

In 2014 the National Patient-Centered Clinical Research Network, [3] was established as a novel initiative of the Patient-Centered Outcomes Research Institute (PCORI), with the goal of accelerating the patient-centered transformation of the culture of clinical research. Patients are considered stakeholders serving in co-PI roles, significantly contributing to the development and management of patient-centered outcomes research (PCOR) [3].

One key approach identified through the PCORI initiative was a new approach to stakeholder involvement building on the PBRN concept and known as Clinical Data Research Networks (CDRNs). CDRNs are the conduit through PCORI for stakeholders to conduct patient-centered research, and focus on the use of data-sharing infrastructure to implement patient-centered research. Thirteen Clinical Data Research Networks (CDRNs) were established starting in 2014 as a strategic model across healthcare delivery sites to provide access to a broad array of clinical data, built on existing electronic health records (EHRs) [4]. The CDRNs are based in multiple types of healthcare delivery sites, including academic and community-based hospitals, clinics, health plans, integrated delivery systems, and federally qualified health centers.

Prior to the development of CDRNs, few studies had illustrated the utility of EHR data to impact health care services, delivery, and outcomes at a population level [5–7]. CDRNs have initiated a paradigm shift synching technological advancements with EHR data. The result is a data warehousing model applied across multiple clinical systems to generate a pool of aggregate and identified use cases primed for pragmatic clinical trials and comparative effectiveness research (CER) [4].

In this paper, we feature the Mid-South Clinical Data Research Network (MS-CDRN), which encompasses three large health care systems: (1) Vanderbilt Health System with electronic medical records for over 2 million patients, (2) the Vanderbilt Healthcare Affiliated Network (VHAN) which currently includes over 45 hospitals, 300 ambulatory practices, and over 3 million patients in the Mid-South, and (3) a partnership with Greenway Health and other national organizations to provide access to over 1600 practices and 18 million patients across the country and (4) the Carolinas Collaborative which includes University of North Carolina at Chapel Hill, Duke University and Health Sciences South Carolina and reaches over 9 million patients.

These health systems in the southeast United States are premised upon the vision of PCORI; “patients and clinicians meaningfully involved in developing and sustaining a thriving CDRN” [1, 8–10] Yet, gaining meaningful involvement from clinicians is a consistent challenge,fueling our study to focus on community clinicians currently underrepresented in research teams [1, 8–10] . Community clinicians primarily deliver patient care and lack protected time as a clinician researcher on a project team.

Our study sought to understand the viewpoints of healthcare providers in the Southeastern United States towards CDRNs and research participation. Study objectives included: (1) identifying and understanding barriers to clinicians’ involvement in MS-CDRN governance, (2) gauging clinician interest in generating research questions and participating in research studies, (3) engaging clinicians in MS-CDRN topic generation and priority setting, and (4) developing strategies and policies to ensure that clinicians are involved in both governing and using the MS-CDRN.

## Methods

### Study design

We employed a mixed-methods sequential explanatory study design [11]. This design features collecting and analyzing quantitative and then qualitative data as two consecutive phases in one study [12, 13]. First, the self-administered quantitative survey data were collected. Sampling for the survey was purposive to represent a range in provider types, practice locations and demographic diversity. This manuscript reports only on the qualitative portion of the study to ascertain clinicians’ perspectives and thoughts about engaging with a CDRN. Quantitative data and their analysis were refined by administering semi-structured interviews to deepen and confirm clinicians’ perspectives about CDRN involvement.

### Population/sample

A subset of survey participants who completed the quantitative survey agreed to participate in the qualitative data collection, an in-depth interview about barriers and facilitators to research participation and CDRN governance. Verbal consent was obtained before the 20-min interview. Each participant received a $100 gift card as compensation. Institutional review board approval was granted from Vanderbilt University Medical Center.

### Study setting

The study setting includes clinicians who are located at community hospitals and a range of outpatient practices including primary care, specialty practices, safety net facilities that serve diverse populations, and academic health centers across the Southeastern United States.

### Recruitment

The quantitative survey used multimodal recruitment approaches, focusing on clinicians underrepresented in research. Those participating in the interview initially participated in the quantitative survey and agreed to be interviewed (*N* = 60). As with all qualitative studies, the goal was more comprehensive data rather than a larger number of samples. The sampling strategy achieved this goal (Table 1).

**Table 1:** Clinician Demographics

	Total (n = 60)
	N (%)
Sex (n = 60)	
Male	25 (41.7)
Female	35 (58.3)
Race (n = 60)	
Caucasian	48 (80.0)
African American	3 (5.0)
Asian	7 (11.6)
Middle Eastern	1 (1.7)
Native American/Alaskan Native	1(1.7)
Discipline (n = 60)	
Physicians	24 (40.0)
Nurses/Nurse Practitioners	18 (30.0)
Dentists	1 (1.7)
Physician Assistants	5 (1.5)
Pharmacists	1 (1.7)
Practice Administrator	1 (1.7)
Dental Hygienist	3(5.0)
Nutritionist	6 (10.0)
Psychologist	1(1.7)
Practice (n = 60)	
Hospital-based	17 (28.3)
Solo	9(15.0)
Single Specialty	12 (20.0)
Multi-Specialty Group	10 (16.7)
Academic Medical	8 (13.3)
Community Health Center	2 (3.3)
General Practice	1 (1.7)
Home Health	1(1.7)

### Procedures/ data collection

A semi-structured interview guide was adapted from an interview guide previously used in understanding participation in Practice Based Research Networks (PBRNs) [14–16]. A group of researchers with experience in both PBRNs and CDRNs selected a subset of questions with the greatest relevance to the CDRN setting from the PBRN interview guide.

The interview for this research sought to capture in-depth feedback from community clinicians regarding their potential participation, usefulness, interest, and attitudes toward a CDRN.

The interview guide included demographic questions, clinic characteristic questions, framing questions to identify if and how long the clinician had been engaged in research and questions with specific prompts to elicit facilitators and barriers to CDRN participation (See Additional file 1).

### Analysis

The interviews were recorded, transcribed, and analyzed using a qualitative content analysis approach [17]. We uploaded interview transcripts and participant characteristics to Dedoose, a cloud-based mixed methods data analysis package [18]. Participant descriptive characteristics included details such as prior research involvement, role (e.g., physician, nurse), specialty (e.g., primary care), practice type, and race/ethnicity. Participant descriptive characteristics were assigned as descriptors to individual interview transcripts, allowing cross-analyses and comparisons across specific characteristics. One researcher (KMU) coded the transcript and descriptive data in Dedoose through a series of iterative coding cycles, which identified interconnected concepts and related codes that produced high-level themes. The research team (KMU, JF, AF) met multiple times to discuss the iterative coding process and evaluate the resulting code tree for relevance to the data.

## Results

The 60 Community clinicians interviewed were 58% (35/60) female, 80% (48/60) Caucasian, 40% physicians, (24/60), 30% (18/60) nurse/nurse practitioners (Table 1). Cross-analysis of the results based on descriptors yielded no significant findings, in part due to sample size. Based on analysis of interview data, results were grouped into 5 themes. The five themes are summarized in Table 2, highlighting the key concepts and concerns voiced by providers. Each of the five themes is described in greater detail below, with illustrative quotes as exemplars of the range of data identified through the iterative coding process.

**Table 2:** Overview of Themes and key concepts

Theme	Key concepts
Theme 1. Research with relevance and benefits to clinics and provider’s patient population	• Clinicians motivation for getting involved with research linked to relevant clinical research studies for patient conditions (i.e. Diabetes, Hypertension) and practice environments
Theme 2. Difficulties of engaging in research with existing patient care demands	• Barriers to research involvement: • Challenges starting research studies • Consequences of time spent on research versus patient care • Substantial pressure on clinicians to maximize the number of patients seen in their practices (“practice efficiency model”) mismatch between clinician reality of integrating research into practice environments.
Theme 3. Clear and continuous two-way communication about research, coordinated with provider and clinic needs	• Clear communication and collaboration as key to setting the tone of a research relationship often related to logistical support of running a clinical practice.
Theme 4. Tailored compensation approaches meet provider preferences	• Different compensation models for research involvement depended on the individual, their role, and their practice model.
Theme 5. Increasing clinician awareness about Clinical Data Research Networks (CDRNs)	• Governance roles and opportunities available in a CDRN, (i.e. who can and should be involved in network governance, time demands of different types of governance involvement, and what CDRN governance means) was not well understood.

### Theme 1

#### Research with relevance and benefits to clinics and provider’s patient population

Regardless of role or specialty, clinicians overwhelming expressed their motivation for getting involved with research was to directly benefit their practices’ patient population.

One physician noted,

*“The patient that we see on a daily basis in the practice, like diabetes, hypertension, hyperlipidemia, emphysema, and COPD [chronic obstructive pulmonary disease]. So, I would more interested in this type of research, the things I see every day…”*

An administrator described quality improvement research as most relevant to their practice:

*“I think our primary care practices benefit most from quality improvement research projects. For example, one of our most recent projects is automatic blood pressure monitoring. We are involved in diabetes monitor controls. We have done smoking cessation research. So, that quality-based translational research is what benefits us most.”*

The care areas described by this administrator—blood pressure monitoring, diabetes management, and smoking cessation—are all common in primary care environments. Linking relevant studies to both practice environments and patient conditions was reiterated by many interviewees.

The clinicians stated they were inherently familiar with the characteristics and needs of their local patient population. This insight allowed them to select practice-specific research studies more aligned with their community of patients. One nutritionist described the importance of local knowledge to research participation:

*“I'd like to have a say in what we selected for research because I would want it to be useful for our patients' outcome, and we know our patients more than the whole system would, or our population, I guess, because that may be different from even a system who is 20 miles away.”*

Many interviewees described tradeoffs in time and effort. Having potential benefits for their patients provided an additional incentive to conducting research. As one physician described,

*“Currently at our practice we have been offered different studies, and the time it would take for us to do them for the benefit we see for the patients, it just isn't worth it… We have had some really good studies come through that we definitely see the benefit for the patient, so we take that extra time to try to recruit.”*

Clinicians derived satisfaction matching patients with relevant studies to improve health or connect to new care pathways. One nurse practitioner described this as a reason for involvement in research:

*“I like identifying the patients and bringing them around to the studies, especially if I think it is going to help them clinically, especially people that may be frustrated with their disease process and they are looking for anything else out there that helps them to feel actively involved, and sometimes even just feeling like they are going outside the box gets them doing the basic things that they had been omitting that could help to improve themselves.”*

Clinicians expressed less interest in the theoretical benefits of research involvement. Several respondents mentioned contributing to the greater good and the body of medical knowledge. Overall, clinicians placed greater emphasis on the direct benefits of the research for their clinical environments and their patients.

### Theme 2

#### Difficulties of engaging in research with existing patient care demands

Clinicians discussed many barriers to research involvement: challenges of getting started in research, consequences of time spent on research, and patient-related barriers to expanding research.

While increasing involvement in relevant research studies appealed to many clinicians, starting in research activities was not always easy. One nurse practitioner described previous experience in research-intensive settings as making her more comfortable participating in research:

*“I was trained at [one research-intensive academic medical center] and then went into [second research-intensive academic medical center], and then of course me working in clinical research, it might be an interesting note to have that, because maybe I think differently because I have been around it for so long, so it doesn't seem that unattractive to me. But I think for a lot of people that didn't train in that type of setting or haven't been exposed, sometimes the idea of it is overwhelming.”*

Most interviewees did not express the concept of comfort level with research, instead they spoke of various levels of familiarity with research processes and expectations. Participants with greater previous exposure to research studies conveyed clearer understanding of time, effort, and materials requirements for research.

Clinicians described the substantial pressure they faced to maximize the number of patients seen in their practices. Participants who described their work environment as being on a “productivity model” voiced concerns about the impact of research activities on efficiency and throughput.

As one physician’s assistant described,

*“The hard thing for us is that our network is driven by productivity and we are incentivized due to productivity, so there would have to be allowances made for that. It wouldn't just be about us making space on a day-to-day basis, but also somehow making up the productivity that is lost in not seeing as many patients in exchange for doing the research.”*

The additional effort of research involvement was not a matter of significant time requirements, but, one participant described it as *“the extra few minutes you spend with a patient.”* One physician described the challenges of a practice efficiency model as *“just trying to keep our heads above water.”* This impact was detailed by one nurse practitioner, who described the different sources of time pressure in her day:

*“You have a full schedule of patients and generally that is not all you are going to have. Perhaps you have 3 or 4 students following you as well. The patient is going to be primary, they are going to be secondary, and research is going to take a back seat to that sort of thing. If you ever got a day to yourself just seeing your patients in the clinic, you could dedicate more time to that [research], but honestly, it's more like survival some days.”*

Clinicians explained the significant time burdens they already face, beyond being involved in research. As one physician stated,

*“If we have 22 patients a day, plus 22 patients with results and letters and phone calls, we have 2 hours at the end of the day of clinical paperwork to do, so where is the time to recruit the patients and call the people and go to meetings?”*

One nurse stated this succinctly: *“We can’t keep up with what we are doing now, let alone adding one more thing to my day.”* Balancing routine work with the research-related work impacted more than the individual clinician and affected practices as a whole, including administrative staff. One physician described frustration with balancing research activities with routine clinical work,

*“Where I used to work, there is a lot of emphasis on trying to get research done, and I would try to help when I could when people would bring up stuff or some colleagues would say, ‘Hey, we are trying to do this kind of study or we are trying to collect this kind of data,’ and it's great and all and I really try to do it, but it got very frustrating, because they just kind of expected it to all just happen within the framework of our 12-hour days and it just kind of added time to our days. The staff got frustrated because they were trying to get involved and had more paperwork to give out, and everyone was already so rushed. So, it just became a negative experience a lot of the time, which changes how it all feels on a day-to-day basis.”*

Clinicians described a mismatch between concepts of time for research involvement and clinician reality of integrating research into practice environments. Clinicians noted that several aspects of research are underspecified (e.g., the time to learn the basics of a study and understand its relevance to patients). One physician explained these education-related time requirements:

*“It's time. It's my time ... being able to set aside time to adequately explore what the trial means to the patient so that they would express interest in being recruited. That's one barrier. Then it's the time it would take for me to become familiar with the ins and outs of the study, sitting down with whoever is leading the trial to find out exactly what they are looking for, which patients would or would not be good candidates to being in the trial, the safety concerns I would need to counsel my patient on. So, you could put that under the closet education for me, but it is really more time. It is just time and being willing to part with my time. I have a family and it is very, very important.”*

Getting involved with research required more than just the time study needs, but the time to understand the basics of the study to recruit the right patients and to adequately explain the study to patients.

Most barriers explored by clinicians focused on clinician and practice-related issues, one nurse practitioner mentioned recruitment challenges.

*“Patients often look at research as ... some of them are really interested, especially if they come from a science background, but a lot of them look at you like you are just looking for a ‘guinea pig.’ That is something I hear fairly often. ‘This is [research-focused academic medical center]. You research people are looking for guinea pigs.’ There is a little mistrust in even the word ‘research.’ Sometimes I word it as, ‘This would be a way you could help other patients.’ I never say the word ‘research,’ and that seems to sometimes help. They think of lab rats, I think.”*

The need to explain a study to patients who are skeptical about research adds yet another layer onto the time requirements of research involvement taking time from routine clinical practice.

### Theme 3

#### Clear and continuous two-way communication about research, coordinated with provider and clinic needs

Clinicians described many facilitators that could help them and their practices become more involved in research. These facilitators included communication, collaboration, additional resources such as staff, appropriate compensation for research involvement, and finding ways to embed research into the existing practice workflow.

A component discussed across multiple roles was clear and unambiguous communication between researchers and clinicians, to develop a sense of collaboration. Clinicians described frustration with communication practices in previous research involvement and the kinds of communication channels needed to overcome these problems. As one nurse practitioner stated,

*“...Things often seem to be sort of off-hand a quick email or verbal, ‘Can you try to help me recruit here or there?’ It is not often that I seem to get an organized, ‘Here are 2 sheets of paper on the criteria we need for this study,’ and more importantly, ‘Here is a way that you can get in touch with me that is not going to be a time constraint for you.’ I need a way to get back to the primary investigators without feeling like a burden.”*

Clear communication and establishing collaboration was described by several participants as key to setting the tone of a research relationship. As one nurse described:

*“You have to have that collaborative effort to make everything go smoothly. That's what you need, and if you don't have that, it will put a damper on what you're trying to do.”*

Consistent communication was sought throughout the study often related to logistical support. One nurse who just wanted information to help with *“…understanding how better to organize it [study materials and data] and get the research done”* and another nurse who described this perspective on research support:

*“I would like to have access to a research coordination center that would have infrastructure available to handle things like taking care of the database, data question issues, database development, study drugs, organization, and shipping…”*

Interviewees also wanted to know more about study progress in a general sense. One physician described ongoing periodic communication about study status by stating,

*“I think that certainly at the midway point of the study, it would be very helpful to see midway point data, and certainly any adverse events that happen.”*

Clinicians discussed potential roles for additional research staff within their practice. One physician described some tasks for a research assistant:

*“[I]f there is follow-up necessary (in other words, contacting patients, having them come back, or follow-up tests or blood work or something like that), it is just very labor-intensive, so we want to make sure we are not asking our already-busy nursing staff and front office staff to have to pick up any of that. So, the barrier I would see is bringing in a research assistant that you can rely on doing the bulk of that work.”*

Ideas for support staff roles and responsibilities varied across interviewees, including administrative tasks and coordination activities, although providers noted the financial implications of additional staff.

*“…it comes down to support staff. I can't do all the paperwork. I can't do all the data entry. I can't chase down a lot of these. We need a good study coordinator that can take care of all the busy work, if you will… So, it usually comes down to funding - you know, how are we going to pay for this?”*

One physician sounded a cautionary note about the need to clearly identify appropriate roles for additional research staff and to understand existing patient-physician relationships to successful research recruitment. The physician stated,

*“The natural conclusion that [the interview study] may reach is - you know, why don't we just pay a nurse to sit in your office and talk with patients that you have identified may be candidates, and that sounds good, because a nurse is certainly cheaper than a doctor's time. But let me tell you this - who has the rapport with a patient? Not a study nurse from [a research-focused academic medical center]. It's the physician who has been looking at the patient all this time. So, unfortunately, if you want to recruit participants for your study, you have really got to compensate the physician for the time they are going to spend with the patient explaining why it is a good idea for the patient to participate in the study. So, you can have the study nurse doing the behind-the-scenes work.”*

The importance of local knowledge, practice experience, and relationships with patients came up multiple times. Although interviewees did not always have a clear plan for what they could contribute to research studies, they explained the value of their local expertise and their existing relationships with individual patients. This concept of local knowledge also proved important when considering how to fit research activities into a specific practice. Interviewees stated that integrating research into routine practice flow was a critical part of successful research involvement. One physician’s idea on this was,

*“The other thing is making sure it is something that really flows and meshes well with the already-planned visits that we have. For instance, we have well checkups, so being able to quickly incorporate that where there is minimal disruption in that already-scheduled normal routine visit. We have been able to do that, but it just can't be too elaborate is the punch line. It just can't be too ambitious of a study for a busy private practice.”*

Another physician succinctly stated, *“We really have to have something that doesn’t interfere with the flow of the office.”*

### Theme 4

#### Tailored compensation approaches meet provider preferences

Hand-in-hand with facilitators for research involvement was that appropriate compensation was needed for individuals and practices to get involved in research. Questions about compensation varied the most among interviewees. The range of responses demonstrated that different compensation models for research involvement depended on the individual, their role, and their practice model. As one physician stated and our interviews supported, *“I think people like choices, and not everyone likes the same choices.”*

While many individuals expressed interest in continuing medical education (CME) credits from research activities, responses to other compensation types varied. The topic of financial compensation demonstrated that no “one size fits all” approach exists. One physician linked compensation to the productivity and efficiency concerns described in Theme 2:

*“I guess it is just really dependent on the nature of what they ask. Most sites in our practice, and probably most practices in many places, don't have much flexible staff time or much space really if there is additional work that would be done at the site. We would probably need something like compensation… if there is any sort of work that would reduce the number of patients we can see in a day, we would need to be compensated for that loss of activity.”*

Interviewees described complexities to financial compensation for productivity model contexts. Some interviewees indicated that direct financial payment for research involvement would not be allowed by their practice group, and that alternate means of financial compensation were necessary. The exact structure of alternate financial compensation was not consistent or clear across interviewees, indicating that this area may require more negotiation and agreement, depending on practice environment and local requirements.

Financial compensation, particularly for community practices, was discussed by multiple interviewees. One physician described this rationale:

*“So, if you are my patient and I wanted to enroll you in a study on diabetes… you and I might talk for 15 or 20 minutes, and that would be time I could spend with another patient, for which I am reimbursed for that time. So, that's why really the biggest thing for community practitioners... the reason a lot of us don't participate in research is because we don't receive a stipend from an academic facility. We receive only reimbursement from patients and insurance companies for what we provide. So, if you are going to spend time recruiting patients into a study, that loss needs to be offset with financial compensation, or there is truly no incentive, except for altruism, to participate in a research study.”*

Beyond financial compensation, interviewees discussed compensation related to publicly acknowledging their research involvement. One nurse stated, *“I would love to have a voice…”* noting the limited involvement of nurses in research. Another nurse brought up being acknowledged in publications and other communications. This nurse described the value of this form of acknowledgement:

*“I don't know what type of recognition you could get, but if you do a clinical trial, and if you have some positive outcomes, to where you have the recognition of saying, ‘We participated in this, and we were able to get this out,’ whether something is published ... and just the idea of seeing if possibly what you did is being put into use. That would be an incentive right there.”*

Authorship of research publications was also discussed by several interviewees. Views of authorship varied across clinicians, depending on practice environment and personal goals. One physician stated,

*“I am a community endocrinologist, so this is the path I have chosen. I haven't pursued a research-based career. So the idea of being on a manuscript doesn't hold nearly as much draw for me.”*

In contrast, another physician described authorship as an incentive:

*“From a personal standpoint, the thing that incentivizes me would be to be involved in writing the paper and publication. That is kind of my goal behind doing this. One is to participate, but I don't want to just be a worker bee. I'm not saying I have to be the first author on the paper, but maybe be a part of the publishing of the study once it has been completed.”*

These contrasting views of the clinicians illustrate the need for individualized and context-aware models of compensation for research involvement.

### Theme 5

#### Increasing clinician awareness about clinical data research networks (CDRNs)

Clinician educational needs tied to the design and procedures of studies and the educational needs of patients about research in Theme 2. However, a broader topic was discussed by many interviewees about understanding what is involved in a CDRN and how to participate.

Interviewees were asked about participating in the governance of a CDRN, which spurred a degree of confusion around what a CDRN involves and what “being involved in governance” entails. As one physician stated,

*“I don't have a good sense of what that [CDRN governance] would actually be about… I understand the words you are saying, but I don't know that I am clear on how that would work.”*

While interviewees uniformly wanted to select research studies for their practice to engage in, other topics about governance were less clearly understood, such as roles and opportunities available in a CDRN, who can and should be involved in network governance, time demands of different types of governance involvement, and what CDRN governance means.

## Discussion

Our CDRN counterparts have veritable lessons learned on engaging clinicians in CDRNs [19, 20]. Their parsimonious approaches use scalable networks, master sharing agreements and embedded studies in clinical settings without disrupting the provision of quality healthcare [21–23].

Our interview study provides insight into clinician perspectives on the MS-CDRN, indicating motivations and challenges to meaningful involvement in research governance.

Study participants were located in the southeastern United States, represented diverse healthcare roles, a range of clinical practice environments, models and structures. Despite the range of participant characteristics, our interviewees indicated that the main motivating factor for getting involved in research was altruistic; wanted to participate in research that was relevant and had the potential to deliver direct benefit for their patient population. Interest in research was balanced by concerns about time and effort required by research activities, particularly when juxtaposed against patient care responsibilities.

Compensating providers for research activities was stated as a clear requirement. Responses from our study participants indicated that flexible strategies instead of a “one size fits all” approach to compensation is required to meet healthcare provider needs. Compensation for CDRN participation needs to have multiple layers, including recognition of contributions, CME credits, and direct financial compensation.

Offering solutions to barriers hindering clinician participation in research, such as flexible compensation models, allows for sustainable engagement that can facilitate significant input increasing the patient-centeredness and practicality of PCORI studies. As the stakeholder engagement continues to gain popularity in the clinical research enterprise, and especially within CDRNs, this work can inform and guide efforts to maintain effective engagement with clinicians in health care settings. The increasingly valuable role of stakeholders on research teams highlights opportunities for evidence-based practice in research to be more efficiently translated to practice in the healthcare setting.

An important insight that emerged was that providers are very interested in research involvement, but are less clear on what network governance might entail and how to get involved in governance in appropriate ways. Clinicians indicated that CDRNs are still relatively new to them. Although not all clinicians in our study wanted the same degree of governance involvement, education and training on what exactly network governance means and what roles are available is crucial to promoting widespread governance participation.

Our research is unique and to our knowledge, no other health system in the CDRN has examined clinician perspectives to engaging in a clinical data research network. Similar research has been conducted in primary care practice based research networks (PBRNs) [23] employing qualitative methods to assess perceived barriers that hamper the participation.

of community medical practices in clinical research, and the perceived facilitators for conducting research in such practices [14, 24, 25]. Several themes emerging from these studies aligned with our findings; perceived barriers to participating in research network; 1) the cost of physician and staff time [14, 24] 2) diversion of research from other clinical tasks [25] and 3) limited time and competing clinical priorities [25]. Likewise, perceived facilitators that supported our findings were 1) interest in clinical research relevant to improving quality of care in the clinical practice or community and 2) Monetary or academic incentives [14].

Contrary to our findings in the CDRN setting, community distrust of research was identified as a perceived barrier by the clinicians to PBRN participation. However, many clinicians felt that if the research was highly relevant to the community and was supported by study materials appropriate in language and literacy, that the trusted relationship between clinician and patient would outweigh barriers to clinician’s participation in research networks. [14, 26]. This could also potentially illustrate a difference between how physicians perceive the use of data as a research tool as compared to more intensive interventions or data collection involving biological specimens.

### Study limitations

All study participants were located in the southeastern United States, and there may be regional differences in how healthcare providers perceive CDRNs. Comparing responses of this provider sample to CDRNS in other regions could yield interesting insights into distinct regional requirements and general CDRN development principles. Data from this study were analyzed by an individual (KMU) not involved in design of the semi-structured interview instrument or in data collection. While the data were analyzed primarily by one individual (KMU), the analysis process and outcomes were discussed in detail to reach agreement about codes with other members of the study team (JF, AF). Finally, the semi-structured interview instrument allowed limited probing of interviewee responses beyond the structured questions. Clinicians may have additional insights that the interview questions did not fully capture. We employed the sequential explanatory model to combat the limitations of solely using the semi-structured interview data for analysis. This mixed-method design has the advantage of exploring quantitative results in more detail through the lens of the qualitative data analysis to refine and exploring participants’ views in more depth [16].

## Conclusion

Providers expressed the most interest in getting involved with research that was relevant and could provide direct benefits to the unique patient population of their practices. Multiple barriers prevent providers from increasing involvement in research studies. Some of these barriers were direct, such as lack of trust in research by their patient population, but others were more intricate, such as complexities of inserting research activities into the practices’ existing workflow. Providers identified various factors that could facilitate additional research involvement including communication, collaboration, and thoughtfully deployed support staff.

No single ideal model for compensation was identified across our study participants. Instead, different compensation approaches were identified for both personal needs and practice-specific requirements. Although some educational needs relate directly to specific research, some also relate to the structure and functionality of a research network.

Recommendations.

A need for clear definition and awareness of a research network and the related governance for providers underrepresented in research is vital. Future research can build on this study by exploring the efficacy of models to increase clinician representation in CDRNs and CDRN governance.

## Additional file

Additional file 1: Qualitative Interview for Providers- Interview and demographic questions posed to clinical providers in the Southeastern United States, Tennessee and North Carolina. (DOCX 28 kb)

## Abbreviations

CDRN: Clinical Data Research Network
CER: Comparative Effectiveness Research
CEnR: Community Engaged Research
CME: Continuing Medical Education
COPD: Chronic Obstructive Pulmonary Disease
EHR: Electronic Health Records
MS-CDRN: The Mid-South Clinical Data Research Network
PBRN: Practice Based Research Network
PCORI: Patient-Centered Outcomes Research Institute
