# Supplementary material for: Clinicians’ perspectives on and interest in participating in a clinical data research network across the Southeastern United States
Source: BMC Health Serv Res. 2018 Jul 20;18:568. doi: 10.1186/s12913-018-3399-9 (PMC6053753; doi:10.1186/s12913-018-3399-9)
Supplement: Supplementary file 1 — Qualitative Interview for Providers- Interview and demographic questions posed to clinical providers in the Southeastern United States, Tennessee and North Carolina. (DOCX 28 kb) [file 12913_2018_3399_MOESM1_ESM.docx]

**Additional File 1**

**Qualitative Survey Interview Demographics**

- 1. What is your race or ethnicity? (check all that apply)
     - Asian (i.e. Asian Indian, Chinese, Filipino, Japanese, Korean, Vietnamese, Hmong, Laotian, Thai, Pakistani, Cambodian, etc.)
     - Black, African American, African, or Afro-Caribbean (i.e. African American, Haitian, Nigerian, etc.)
     - Hispanic, Latino, or Spanish origin (i.e. Mexican, Mexican American, Puerto Rican, Cuban, Argentinian, Colombian, Dominican, Nicaraguan, Salvadorian, Spaniard, etc.)
     - Middle Eastern/North African
     - Native American, American Indian or Alaskan Native (i.e. Navajo, Mayan, Tingt, etc.)
     - Native Hawaiian or Other Pacific Islander (i.e. Native Hawaiian, Guamanian or Chamorro, Samoan, Fijian, Tongan, etc.)
     - White (i.e. German, Irish, Lebanese, Egyptian, etc.)
     - Some other race or origin (please specify)_
     - Prefer not to answer
  2. What is your sex?  Male  Female  Other  Prefer not to answer
  3. What is your discipline/role?

Physician  Nurse practitioner  Nurse  Pharmacy  Physician Assistant  Dentist  Nutritionist

Physical/Occupational/Respiratory Therapist  Psychologist, Licensed Social Worker  Administrator

- - - Other Clinical Care Field (If other clinical field please describe)
  1. Which best describes your practice?

| - Solo practice | - Single specialty group | - Multi-specialty group |
| --- | --- | --- |
| - Community Health Center - VA practice | - Hospital based practice - Other If other please describe | - Academic medical practice |

- 1. Are you affiliated with any of the following hospitals? (Check all that apply)
     - Vanderbilt Univ. Medical Center  Nashville General Hospital at Meharry  Saint Thomas West Hospital
     - Saint Thomas Midtown Hospital  Skyline Medical Center  Centennial Medical Center
     - West Tennessee Healthcare  Williamson Medical Center
     -  Maury Regional Medical
     - Cookeville Regional Medical Ctr  Mount States Health Alliance
     - Other___________

**CDRN Semi-Structured Interview questions: Provider Questions**

**Introduction:**

You have been identified by our study team as a provider who actively engages with patients or as an individual that contributes to the decision making in your clinic or health system. We are interested in your thoughts and input on the needs and barriers to participating in a clinical data research network. We would like to learn about what type of environment exists at your medical practice and gauge the interest level of your practice in participating in a research network. A research network is defined as “a data network that has pooled de-identified records of willing patients with records from other practices in the network to enable patient centered clinical research studies”

**Verbal Consent:**

In order to develop the infrastructure of a clinical data research network we would like to get input from providers/stakeholders/clinicians regarding their potential participation, usefulness, interest and attitudes towards a research network. I will be asking you a series of questions to better understand your perspective on involvement in a research network. Please be open and honest with your responses. This interview will be audio-recorded. If you would like to stop the interview at any point in time, feel free to do so. Audio recordings will be transcribed and destroyed thereafter. You will receive $100 for participating in this interview. This will serve as a verbal consent, do you consent to moving forward?

**Background questions:**

1. 1a) Are you currently involved in research? 1b) Have you been involved with research in the past?

**[if the answer to Question 1a. is “YES” skip to Question 2a. if the answer to Question 1a. is “NO” proceed to Question 1b.]**

**[If the answer to Question 1b is “NO,” skip the entire Question 2 and proceed to Interview question 1.]**

1. 2a) How many months/years did you conduct research? 2b)What type of research did you conduct?

**NOTE: Before beginning the interview, reiterate the importance of answering the question exactly the way it is asked to them. For example, if asked to define a research network, refer to the highlighted definition above. If asked to define another term like barriers, ask the participant what barriers means to them. This technique will facilitate things more smoothly and not bias the respondent’s answers.**

**Interview Questions (parenthesis indicate more of a PBRN approach to the questions):**

1. If you (your practice) had all the resources you (it) needed to participate in research, what types of research studies would you be most interested in becoming involved with and how active would you want to be?

Prompts: Randomized clinical trial, Observational study, Case study, others?

**[If they ask for examples of research studies, give them the examples in the “prompts” section]**

1. What types of infrastructure/support needs would you (your practice) need to make participation feasible?

Prompts: additional space, additional personnel, more flexible EMR, technical support, educational training, MOUs, dedicated research staff, more time in the day, others?

**[Be prepared to explain that “MOU” stands for Memorandum of Understanding, if you use it in the prompt section.]**

1. What types of incentives would you (your practice) need to make research participation desirable to you (your practice)?

Prompts: financial reimbursement, MOCs and CME/CEUs, additional training, clinical updates, ability to co-author manuscripts, scholarships to attend conferences to co-present findings

**[“MOC” stands for Maintenance of Certification. “CME” stands for Continuing Medical Education, which are educational credits for physicians. “CEU” stands for Continuing Education Units, which are educational credits for Non-Physicians, usually staff and personal. ]**

1. What type of barriers would prevent you (your practice) from participating in a research network? Prompts: lack of staff, demands of clinical practice, type of study designs offered not applicable to patient’s eligibility, level of participation as investigator too demanding, different levels of comprehension of research process by clinicians ( < 1 year vs. > 10 experience)

**[Remember to have the participant define barriers in their own terms]**

**[If they ask “what types of barriers,” use the prompts to provide a few examples.]**

1. What level of governance/input would you (your practice) want to have if you became a member of a research network?

Prompts: right to select which studies to become involved with on a case-by-case basis, active vote in prioritizing which studies network devoted time to, seat on governing body for network, Making eligible patients aware of study availability, helping recruit patients into a study, Referring patients for experimental procedures, etc.
